# Statin-Induced Nitric Oxide Signaling: Mechanisms and Therapeutic Implications

**DOI:** 10.3390/jcm8122051

**Published:** 2019-11-22

**Authors:** Armita Mahdavi Gorabi, Nasim Kiaie, Saeideh Hajighasemi, Maciej Banach, Peter E. Penson, Tannaz Jamialahmadi, Amirhossein Sahebkar

**Affiliations:** 1 Research Center for Advanced Technologies in Cardiovascular Medicine, Tehran Heart Center, Tehran University of Medical Sciences, Tehran 1411713138, Iran; armitamahdavi61@gmail.com (A.M.G.); breeze.nasim@yahoo.com (N.K.); 2 Department of Medical Biotechnology, Faculty of Paramedicine, Qazvin University of Medical Sciences, Qazvin 1531534199, Iran; saeideh.ghasemi67@yahoo.com; 3 Department of Hypertension, WAM University Hospital in Lodz, Medical University of Lodz, Zeromskiego 113, 93-338 Lodz, Poland; maciej.banach@icloud.com; 4 Polish Mother’s Memorial Hospital Research Institute (PMMHRI), 93-338 Lodz, Poland; 5 School of Pharmacy and Biomolecular Sciences, Liverpool John Moores University, Liverpool L3 3AF, UK; P.Penson@ljmu.ac.uk; 6 Halal Research Center of IRI, FDA, Tehran 1411713138, Iran; jamiat931@mums.ac.ir; 7 Department of Nutrition, Faculty of Medicine, Mashhad University of Medical Sciences, Mashhad 9177948564, Iran; 8 Biotechnology Research Center, Pharmaceutical Technology Institute, Mashhad University of Medical Sciences, Mashhad 9177948564, Iran; 9 Neurogenic Inflammation Research Center, Mashhad University of Medical Sciences, Mashhad 9177948564, Iran; 10 School of Pharmacy, Mashhad University of Medical Sciences, Mashhad 9177948564, Iran

**Keywords:** statin, endothelial nitric oxide, angiogenesis, heart failure, atherosclerosis, cancer, neuroprotection

## Abstract

In addition to their cholesterol-lowering effects, statins are associated with pleiotropic effects including improvements in heart failure (HF), reduced blood pressure, prevention of the rupture of atherosclerotic plaques and improved angiogenesis. In addition to these cardiovascular benefits, statins have been implicated in the treatment of neurological injuries, cancer, sepsis, and cirrhosis. These cholesterol-independent beneficial effects of statins are predominantly mediated through signaling pathways leading to increased production and bioavailability of nitric oxide (NO). In this review, the mechanistic pathways and therapeutic effects of statin-mediated elevations of NO are described and discussed.

## 1. Introduction

Statins reduce the endogenous synthesis of cholesterol by inhibiting hepatic hydroxymethylglutaryl (HMG) CoA reductase, the rate-limiting step of the mevalonate pathway of cholesterol production. This causes an upregulation of hepatic low-density lipoprotein (LDL) receptors and decreased deposition of LDL particles in the vascular walls, a necessary event in the pathogenesis of atherosclerosis. Pleiotropic effects of statins make this drug class distinct from other lipid-modifying agents [1,2] are those effects independent of cholesterol-lowering. Numerous putative therapeutic benefits have been ascribed to statins including suppression of apoptosis, antioxidant and anti-inflammatory effects, immunomodulation, neuroprotection and promotion of tissue regeneration [3,4,5,6,7,8,9]. Several of these effects are mediated through the actions of statins on endothelial nitric oxide (NO) signaling pathways. 

Several studies have confirmed that statins upregulate the production and activity of NO [10,11]. Stains including lovastatin [12], simvastatin [13], atorvastatin [14], rosuvastatin [15], fluvastatin [16], pravastatin [17], and cerivastatin [16], increase NO through a variety of mechanisms and pathways, which increase the expression and function of endothelial NO synthase (eNOS). For example, 14 days of exposure of mice to atorvastatin has been shown to evoke an approximately three-fold increase in the expression and activity of eNOS [14]. Statins also increase bioavailable NO by preventing the elimination of NO by reactive oxygen species (ROS) [18,19]. 

In this review, we will discuss the molecular mechanisms and signaling pathways by which statins can modulate NO, and thereby exert cholesterol-independent pleiotropic therapeutic effects.

## 2. Signaling Pathways and Molecules Mediating the Effect of Statins on NO Production

**Figure 1:**
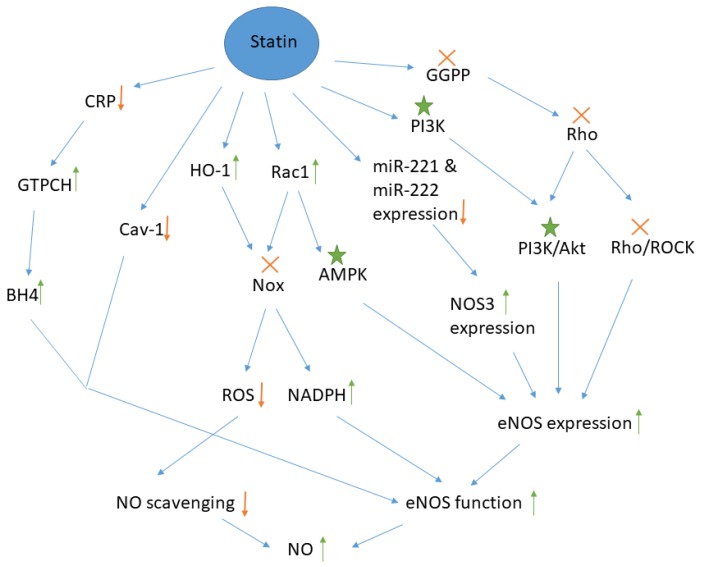
Molecules and pathways involved in the effect of statins on NO production by endothelial cells. 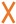: Molecule has become unavailable or inactivate or the signaling pathway is suppressed, 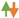: Increase or decrease in the amount, 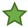: Molecule is activated. GGPP: geranylgeranyl pyrophosphate, ROCK: Rho-associated protein kinase, PI3k: Phosphoinositide 3-kinase, Akt: Protein kinase B, AMPK: AMP-activated protein kinase, miR: micro RNA, HO-1: Heme oxygenase 1, BH4: tetrahydrobiopterin, Nox: NADPH oxidase, Cav-1: caveolin-1, GTPCH: guanosine-5-triphosphate cyclohydrolase, CRP: C reactive protein, NO: nitric oxide, eNOS: endothelial NO synthase, ROS: reactive oxygen species. It is noteworthy that Rac1 is actually a downstream signal for GGPP and the statin-mediated inactivation of GGPP reduces Rac1 level but as statins exert a direct inductive effect on Rac1, the up-regulation of Rac1 and its downstream signaling are shown separately here.

Statins can influence many signaling pathways and molecules which regulate the production of NO by endothelial cells. The major pathways are summarized schematically in Figure 1. Statins increase the expression of eNOS, mainly through inhibition of the Rho/ROCK pathway or by activation of either the Phosphoinositide 3-kinase (PI3k)/ Protein kinase B (Akt) or the AMP-activated protein kinase (AMPK) pathways. Inhibition of mevalonate synthesis by atorvastatin, simvastatin and lovastatin causes upregulation of Rho GTPase expression in the cytosol. The result of Rho GTPase upregulation is inactivation of Rho, because statins make isoprenoids such as geranylgeranyl pyrophosphate (GGPP) unavailable for isoprenylation and post-translational modification of Rho protein, thereby interfering with translocation of this protein from cytosol to the membrane and its activation. Inactive Rho inhibits the Rho/Rho-kinase (ROCK) pathway, which leads to an increase in the half-life of eNOS mRNA and increased production of NO [13,14,20].

The mechanism of PI3K/AKT activation by statins is not fully understood, but it is known that PI3K phosphorylates, and thereby activates Akt [21]. Phosphorylated Akt stimulates phosphorylation of eNOS and NO production in cultured endothelial cells and endothelial progenitor cells (EPC) [22,23,24]. PI3K inhibitors suppress the effect of statins on Akt, demonstrating that the PI3K/Akt pathway is involved in the effect of statins on NO production [25]. Furthermore, Rho inhibition by statins promotes activation of Akt and conversely, overexpression of Rho leads to inhibition of eNOS activation [13,26]. The mediator of eNOS phosphorylation in the PI3K/Akt pathway is thought to be heat shock protein 90. Tyrosine phosphorylation of this protein by statins facilitates its direct interaction with Akt and drives the formation of an Akt/eNOS complex, which results in eNOS activation [27].

Activation of the AMPK pathway is another mechanism by which statins may enhance NO production. For example, incubation of human umbilical vein endothelial cells (HUVECs) with atorvastatin increased AMPK activity and eNOS phosphorylation in a time and dose-dependent manner [28]. AMPK enzymatically inactivates enzymes involved in cholesterol biosynthesis such as HMG-CoA. Therefore, statins and other inhibitors of HMG-CoA activate AMPK [17,29,30,31,32,33]. Based on in vitro and in vivo studies, atorvastatin, pravastatin, and simvastatin increase AMPK activation via phosphorylation at Thr-172 by increasing the GTP-bound Rac1 which promotes eNOS phosphorylation at Ser-1177. As expected, AMPK inhibitors block the effect of simvastatin on eNOS phosphorylation [17]. Interestingly, activation of AMPK by statins is dependent on the duration of exposure and whilst statin treatment increases AMPK activity after four hours, AMPK activity returns to baseline after 24 h [28].

Increasing the expression of NOS3, a gene encoding eNOS is another mechanism by which statins increase the generation of NO in endothelial cells. However, bioinformatic studies have not identified the target region for these microRNAs on the NOS3 mRNA molecule. Cerda et al. showed that microRNAs such as miR-221 and miR-222 reduce the expression of NOS3. Simvastatin reduces expression of miR-221 and atorvastatin mediates expression of both miR-221 and miR-222 in cultured HUVECs, thereby increasing NOS3 gene expression and eNOS function [34]. NO production could be altered through epigenetic modifications exerted by statins. These drugs change the expression of miRNAs and their corresponding target pathways, namely the Rho pathway, thereby regulating NO release from the endothelial cells [35]. Antioxidant properties are a well-recognized pleiotropic effect of statins [36,37]. The signaling molecules responsible for this effect are also responsible for the effects of statins on NO. Statins inhibit superoxide formation by blocking the activity of the oxidative stress-producing enzyme, NADPH oxidase (Nox), by facilitating membrane translocation of Rac1, a cytosolic subunit of Nox [38]. Wassmann et al. showed that atorvastatin reduced expression of essential membrane-bound subunits of Nox, p22phox, and Nox1 [39]. Simvastatin has also been shown to inhibit expression of Nox1 and Nox2 genes in osteocytes [40]. Both Rac1 translocation and heme oxygenase 1 (HO-1) upregulation by statins affect Nox inactivation. The enzyme HO-1 is an intracellular target for statins, and its upregulation reduces iNOS expression, O2- generation, and Nox availability. Finally, Nox inactivation leads to higher NO bioavailability by two mechanisms. Firstly, Nox inactivation prevent ROS production and NO scavenging by free radicals [18,19], and secondly, lack of Nox activity increases the amount of available NADPH, a cofactor for the synthesis of NO by eNOS from L-arginine in the presence of O2 [41].

Statins increase the concentration of tetrahydrobiopterin (BH4) by two mechanisms. BH4 is a cofactor for eNOS function, and hence increased BH4 may explain increased availability of NO. Statins can increase availability of BH4 by increasing the activity of GTP cyclohydrolase 1 (GTPCH), a rate-limiting enzyme in the synthesis of BH4. This appears to be achieved via a statin-induced reduction in C reactive protein (CRP), a suppressor of GTPCH [42]. Additionally, statins prevent the oxidation of BH4 to BH3. The result of these processes is reduced oxidative stress in endothelial cells and improved eNOS function [43].

One proposed mechanism for the effect of statins on NO, which is still controversial, is the inhibition of eNOS inactivation by caveolin-1 (Cav-1) following statin treatment [44]. Caveolae are distinct regions on the plasma membrane containing cholesterol and caveolin proteins which mediate cell signaling [45,46]. Cav-1 from caveolae inactivates eNOS through its attachment via N-terminal myristoylation and palmitoylation [47]. Cholesterol in the caveolae affects the Cav-1 function so that the exposure of cells to free cholesterol results in up-regulation of Cav-1 expression [48]. Therefore, statins play a role in the Cav-1 downregulation by inhibiting biosynthesis of cholesterol [49]. As a result, eNOS/Cav-1 complexes cannot be formed, and eNOS remains in its active state. Meda et al. transfected HUVECs with Cav-1 siRNA using magnetofection method, to decrease Cav-1 production. Then, NOS activity in the control cell and transfected cells and their subcellular fractions was measured after exposure of cells to statins such as fluvastatin, lovastatin, and cerivastatin. The results showed that these statins increased eNOS activity and NO synthesis in a concentration-dependent manner, which was evidenced by the increased accumulation of intracellular cGMP as an indicator of NO bioactivity. In this study, statins did not alter Cav-1 expression and it was hypothesized that statins instead altered the translocation of Cav-1 [16].

All the pathways and signaling molecules by which statins may elevate NO additionally activate other signaling pathways such as the stromal cell-derived factor 1 α (SDF-1α)/ CXC chemokine receptor-4 (CXCR-4) pathway. It has been shown that a seven-day infusion of atorvastatin in rats suffering from acute myocardial infarction increased eNOS and eNOS phosphorylation, activated the eNOS/NO pathway, and finally increased expression of CXCR4 and SDF-1α [50]. Activation of the SDF-1α/ CXCR-4 pathway promotes EPCs mobilization and differentiation into vessel-forming cells [51].

## 3. Therapeutic Implications of Statin-Induced Elevation of NO

The statin-induced elevation of NO described above has important clinical implications. NO is an important mediator of vasodilatation, it prevents leukocyte adhesion, smooth muscle cell proliferation, and platelet aggregation, and it improves endothelial and vascular function [52]. Improving endothelial function by statins suggests their potential therapeutic effects in cardiovascular diseases such as atherosclerosis [53], hypertension [54,55], and HF [56]. Enhanced NO production by endothelium could also provide protection against ischemia in the brain [57]. Whether the effect of NO is to promote new vessel formation in ischemia or to block angiogenesis in tumors and atherosclerosis plaques may depend upon its local concentration in the tissues of interest [58].

### 3.1. Heart Failure

It has been reported that simvastatin increases eNOS expression and NOS activity in the left ventricle (LV) and aorta of rats [56]. In animal models of chronic heart failure (CHF), treatment with cerivastatin or fluvastatin after myocardial infarction (MI) resulted in better LV function and survival [59,60]. In a randomized controlled trial, oral simvastatin improved myocardial ultrastructure [61]. In CHF patients, flow-mediated dilatation was enhanced after the treatment of patients with rosuvastatin [62]. Some studies have shown that atorvastatin has cardioprotective effects, improves echocardiographic parameters, prevents LV dilatation, and delays the progression of HF. The improved myocardial remodeling by atorvastatin was attributed to reduced CRP and inflammation, and increased angiogenesis in cardiac muscle fibers. These effects are mediated by NO production [63]. Atorvastatin blocks the RhoA/Rho kinase pathway and increases the amount of mRNA for eNOS [64]. Upregulation of eNOS expression by statins increases capillary density and/or improves endothelial function, leading to better myocardial perfusion and improvement of HF [65]. The beneficial effects of statins on cerebral ischemia were not evident in eNOS gene knockout mice, confirming that these effects are mediated by NO [66]. However, there are controversies regarding beneficial effects of statins in HF [67,68,69].

### 3.2. Hypertension

A meta-analysis of randomized controlled trials of statin therapy has shown that statins, including pravastatin and simvastatin reduce systolic blood pressure by about 2-mmHg in both hypertensive and normotensive patients [54,55].

Due to the immediate effects of statin on blood pressure and a lack of relationship with the cholesterol response demonstrated in meta-analysis, the mechanism of this effect has been attributed to increase NO production by endothelial cells, and decreased concentrations of endothelin-1, a potent vasoconstrictor protein [54,70]. NO is a potent endogenous vasodilator which causes vasodilatation through binding the soluble guanylyl cyclase, the enzyme responsible for the conversion of guanosine-5-triphosphate (GTP) to cyclic 3,5-guanosine monophosphate (cGMP) [52]. Additionally, the vasodilator effect of statins results from prevention of the generation of ROS, including hydroxyl radicals [71]. Furthermore, statins decrease arterial stiffness and improve systemic arterial compliance by altering the smooth muscle cells composition in artery walls and by restoring endothelial function [71,72]. Studies have demonstrated that incubating isolated rat mesenteric resistance arteries with simvastatin reduces contractility, increases vessel diameter and results in hypotension in such arteries via NO-dependent mechanisms [17,73].

### 3.3. Atherosclerosis

The pivotal role of NO in atherosclerosis process and the NO elevating effect of statins mean that statins have the potential to modulate the course of this condition independently from their well-characterized lipid-lowering effects. Endothelial dysfunction is an early manifestation of atherosclerosis. In the physiological state, the release of NO from endothelial cells protects the endothelium from platelet adhesion in addition to its vasodilator effects. In the early stages of atherosclerosis, oxidative stress, characterized by impairing NO bioavailability results in endothelial dysfunction. Increased oxidative stress at atherosclerotic site turn O_2_ to ROS such as O2- which readily reacts with NO to form peroxynitrite (ONOO-) or nitrosothiols. ONOO−is a highly reactive, highly toxic, and pro-inflammatory agent that oxidizes and inactivates eNOS [74,75].

Statins have the potential to slow the progression of atherosclerosis through NO-mediated mechanisms, and conversely, the rate of development of atherosclerosis in Apolipoprotein E (ApoE) mice is increased when they lack eNOS [53]. In vitro treatment of injured HUVEC cells as a model of dysfunctional vascular endothelial cells in atherosclerosis showed that rosuvastatin reduced cell apoptosis by increasing NO content [15].

Two distinct mechanisms govern the beneficial effects of statins on atherosclerosis. Firstly, statins activate AMPK, which inhibits Nox phosphorylation and activation by protein kinase C (PKC) [76,77]. The antioxidant properties of statins prevent ROS generation and the formation of ONOO−. For example, simvastatin with L-arginine supplementation reduced ONOO− in hypercholesterolemic subjects [78], thereby potentially preserving eNOS activity. Secondly, statins improve stability of the plaques, thereby making them less prone to rupture. Although the mechanism for this effect is yet unclear, it has been hypothesized that statins induce plaque calcification [79,80]. By restoring the NO/ONOO− balance and preventing the progression of atherosclerosis, statins activate eNOS, increase NO bioavailability, reduce the release of ONOO− from the endothelium, and increase HO-1 expression and total HO activity [81].

A study which included 30 patients with peripheral arterial diseases showed that one-month of treatment with atorvastatin significantly reduced plasma ONOO− levels and reduced oxidative stress, resulting in reduced eNOS uncoupling and increased NO production [43]. Rasmusen et al. also showed that atorvastatin combined with a NO precursor, arginine, exert anti-atherosclerotic actions via eNOS activation and NO production [82].

### 3.4. Sepsis

Sepsis is an inflammatory disease consisting of pathological changes in arteriolar tone and endothelial cell integrity, leading to severe hypotension. Loss of NO, overproduction of inducible nitric oxide synthase (iNOS), and the imbalance of iNOS and eNOS are responsible for this disease. Statins such as cerivastatin, simvastatin, atorvastatin, fluvastatin, and pravastatin, increased survival in experimental sepsis models by restoring the iNOS/eNOS balance. In sepsis treatment, hydrophilic statins have priority over hydrophilic ones due to their higher lifetime and potency [83].

### 3.5. Cirrhosis

Statins have beneficial effects on hepatic disorders such as cirrhosis. In cirrhosis, intrahepatic vascular resistance increases due to endothelial dysfunction caused by impaired NO bioavailability. Simvastatin and atorvastatin have been shown to reduce ROS generation, increase eNOS activity and preserve NO availability. The resultant vasodilation leads to a reduction of portal pressure selectively in the hepatic microvasculature and a reduction of hepatic resistance. These effects have been demonstrated in an in vivo rat model of cirrhosis and confirmed in clinical trials [84,85,86,87].

### 3.6. Therapeutic Induction of Angiogenesis and Vasculogenesis

**Figure 2:**
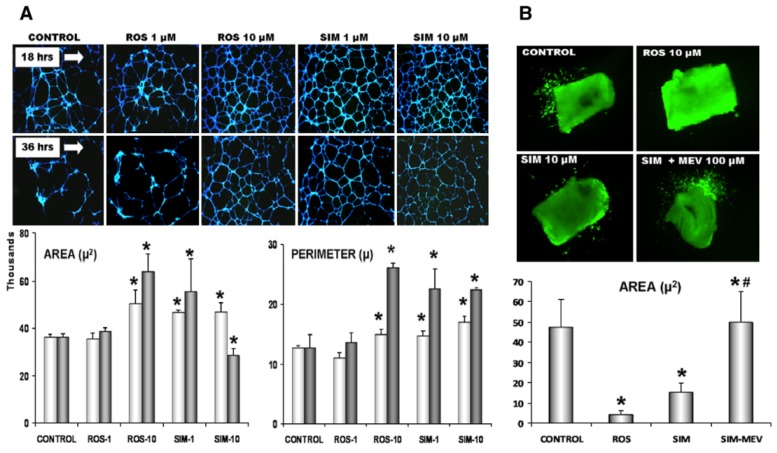
Induction of angiogenesis and vasculogenesis by statins. (**A**) Matrigel angiogenesis assay. HUVECs were exposed to rosuvastatin (Ros) and simvastatin (SIM) at 1 and 10 μM for 18 (upper panel of images) and 36 h (lower panel). Representative images show cells loaded with calcein for fluorescent analysis: Gr *p < 0.05 vs. untreated control. (**B**) Representative images showing the effects of ROS pretreatment with 100 µM L-mevalonate on simvastatin-treated rings; the graph displays simvastatin-treated aortic rings. Data is shown as means ± SE, based on 3–4 independent experiments. Reproduced with permission from [94].

Therapeutic angiogenesis and vasculogenesis are the generation of new blood vessels in conditions resulting from insufficient vasculature, such as ischemic tissues or tissue engineering scaffolds [88]. Statins induce therapeutic angiogenesis and vasculogenesis (Figure 2). For example, rosuvastatin treatment causes circulating EPCs originating from bone marrow to mobilise into the ischemic site of a mouse model of surgically induced hindlimb ischemia, through the activation of Akt/eNOS pathway [89]. A study by Urbich et al. demonstrated that low doses of atorvastatin and mevastatin (0.01 to 0.1 µmol/L) improve the migration of mature endothelial cells and improve tube formation and angiogenesis. These statins also increased vasculogenesis through actions on circulating EPCs. The involvement of both mature endothelial cells and circulating EPCs after statin treatment suggests that statins can induce both angiogenesis and vasculogenesis [89,90,91]. Therefore, statins have been loaded into tissue engineering scaffolds made from mesoporous hydroxyapatite microspheres or titanium to induce angiogenesis in the structure [92,93].

The ability of cells to form tubes after statin exposure is mediated by both the PI3K/Akt pathway (evidenced by suppression of migration by a PI3k inhibitor) and AMPK pathways. Sun et al. showed that the effect of atorvastatin on angiogenesis and tube formation ability of HUVECs is mediated by AMPK activation [28]. Furthermore, statins activate endothelial Ras which activates Akt phosphorylation. Activation of Akt in this pathway leads to posttranscriptional activation of the eNOS. Increased eNOS phosphorylation leads to eNOS/NO pathway activation and NO production. For example, exposure of transplanted mesenchymal stem cells (MSCs) to atorvastatin under hypoxic conditions increased neovascularization in peri-infarcted areas of the heart by upregulating eNOS [95,96]. In another experiment, loading statin into a tissue engineering scaffold designed for regenerating intractable diabetic skin wounds promoted angiogenesis through upregulation of eNOS and NO synthesis [97].

### 3.7. Neuroprotection

**Figure 3:**
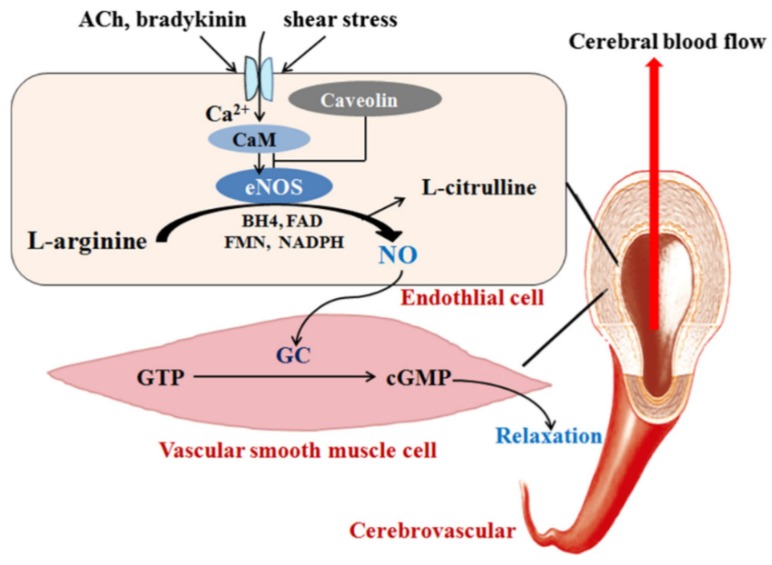
eNOS and its role in the regulation of CBF. eNOS is activated by ACh, bradykinin, shear stress, etc., and then catalyzes L-arginine to generate NO which moves into vascular smooth muscle cells, reacts with GC, and promotes the conversion of GTP into cGMP, resulting in vascular smooth muscle relaxation and the CBF increase. eNOS: Endothelial oxide synthase, CBF: cerebral blood flow, Ach: Acetylcholine, NO: nitric oxide, GC: guanylate cyclase, GTP: guanosine triphosphate, cGMP: cyclic guanosine monophosphate. Reproduced with permission from [101].

Neuroprotection by statins occurs through a variety of mechanisms including reduced expression of the mammalian target of rapamycin (mTOR) protein, increasing brain-derived neurotrophic factor (BDNF) and glial-cell-line-derived neurotrophic factor (GDNF) [98]. Generation of NO by eNOS and nNOS (neuronal NOS) is another mechanism of neuroprotection. NO regulates cerebral blood flow after brain injuries and is a potent neuroprotective factor [57]. The mechanism of cerebral blood flow regulation by eNOS is shown in Figure 3. Therefore, statins are beneficial in the treatment of brain ischemia because they increase the expression of eNOS by inhibiting changes in Rho-mediated actin cytoskeleton [99]. Expression of eNOS is decreased in some neurological injuries, such as strokes and cerebral artery occlusion [57]. In these situations, statins exert neuroprotective effects through restoring eNOS expression. Cerebral blood flow is enhanced by eNOS, stroke severity is reduced and neurological function is improved, as demonstrated by the fact that cerebral blood is impaired in eNOS knockout mice [57,100,101]. Daily injection of atorvastatin to mice for 14 days reduced stroke volume by up to 38% in cerebral arteries by upregulation of type III NOS in aortas and in thrombocytes, and inducing NO production in both the endothelium and also, blood platelets. Thus, platelet aggregation in a thrombus was evidenced by reduced markers of platelet activity, BF 4 and β-TG. Since no alteration in these markers was observed in atorvastatin-treated eNOS knockout mice, the changes in platelet function have been attributed to the increased eNOS expression by statins [12]. 

### 3.8. Cancer Treatment

Statins have demonstrated anti-proliferative and pro-apoptotic effects in cancers. For example, a 40% risk reduction in liver cancer has been attributed to statin use by a meta-analysis [102]. Anti-cancer properties of statins are mediated either by induction of tumor cell cytotoxicity (by enhancing cytotoxic concentrations of NO) or impairing tumor angiogenesis via mechanisms independent of NO [103]. Statins increase NO concentrations through activation of inducible NOS (iNOS) which, in turn, initiates antitumor activity in macrophages and induces down-regulation of the expression of the anti-apoptotic proteins such as survivin. Therefore, transfection of tumor cells with the iNOS gene exerts antitumor effects [104,105]. Kotamraju et al. showed that simvastatin and fluvastatin induce apoptosis in breast cancer cells through production of NO mediated by iNOS so that exposure of MCF-7 breast cancer cells to sepiapterin, an eNOS activator, increases NO synthesis and improves the pro-apoptotic effects of simvastatin and fluvastatin [106]. Statins also have anti-angiogenic properties in malignant tumors through mechanisms attributed to HIF-1α inhibition via AMPK activation rather than NO increase by statins [30].

The concentration of statin is vital in determining whether anti-angiogenic or proangiogenic effects are observed. Low concentrations of cerivastatin and atorvastatin (0.005 to 0.01 µmol/L) improve angiogenesis via eNOS-activation, while high levels (0.05 to 1 µmol/L) decrease angiogenesis—which is useful for prevention of tumor angiogenesis in the treatment of cancer. The angiostatic properties of statins result from induction of apoptosis at high doses and reduction of the release of vascular endothelial growth factor (VEGF) from endothelial cells. Moreover, statin-induced reduction in GGP, a necessary molecule for Rho membrane localization and VEGF receptor activation, contributes to the anti-angiogenic properties of statins [58].

## 4. NO-Releasing Statins to Augment the NO-Mediated Therapeutic Effects of Statin

Introducing a NO-releasing moiety into the statin structure is an interesting idea to add to the NO-mediated therapeutic potentials of statins. For example, adding a NO-donating moiety to atorvastatin improved the ability of this statin to reduced atherosclerosis, inflammation and ROS generation [107]. The amount of released NO affects the effectiveness of these modified statins. Increased nitrosyl hemoglobin formation in the blood can be used to monitor release of NO from the statin structure. Ongini et al. incorporated a nitric ester moiety to the pravastatin and fluvastatin structure and formed more lipophilic statin derivatives that could easily penetrate the cell. Improved cell penetration led to better antiproliferative and anti-inflammatory effects; the former was confirmed by inhibited [3H] thymidine incorporation, and the later was evidenced by inhibited accumulation of nitrite, an oxidation product of NO [108]. Statins induce hepatic and muscular toxicity, as well as severe weight loss in rats suffering from hepatic cirrhosis while NCX 6560, a NO-releasing derivative of atorvastatin, reduced portal hypertension and intrahepatic vascular resistance more effectively than statins without this property of additional NO release [109].

## 5. Conclusions

Many pleiotropic effects of statins, including therapeutic effects in conditions including HF, hypertension, atherosclerosis, sepsis, cirrhosis, cancer, and neurologic disorders are mediated through the impact of statins on NO signaling pathways. Statins increase eNOS expression and activity and reduce NO scavenging by free radicals. Therefore, statins ameliorate NO release and bioavailability. NO is a vasodilator, promotes angiogenesis and neuroprotection, and protects the endothelium from platelet adhesion with the potential to either reduce cell apoptosis at low doses or to increase it in tumor cells at high doses. All these beneficial effects of statins, which are mediated by NO signaling, could be enhanced by modifying the chemical structure of the statin in such a way that statins release additional NO to that produced from activated eNOS. Further evidence from the clinical setting is warranted to better elucidate the interactions of statins with the NO signaling pathways, and the role of such interactions in the cardiovascular, as well as pleiotropic effects, of statins.

## Abbreviations

HMG	Hydroxymethylglutaryl
NO	Nitric Oxide
eNOS	Endothelial NO Synthase
ROS	Reactive Oxygen Species
ROCK	Rho-Associated Protein Kinase
PI3k	Phosphoinositide 3-kinase
Akt	Protein kinase B
AMPK	AMP-Activated Protein Kinase
GGPP	Geranylgeranyl Pyrophosphate
EPC	Endothelial Progenitor Cells
HUVECs	Human Umbilical Vein Endothelial Cells
Nox	NADPH Oxidase
HO-1	Heme Oxygenase 1
BH4	Tetrahydrobiopterin
GTP	Guanosine-5-triphosphate
GTPCH	GTP Cyclohydrolase
CRP	C Reactive Protein
Cav-1	Caveolin-1
SDF-1α	Stromal Cell-Derived Factor 1 α
CXCR-4	CXC Chemokine Receptor-4
LV	Left Ventricle
CHF	Chronic Heart Failure
MI	Myocardial Infarction
cGMP	Cyclic 3,5-guanosine Monophosphate
ApoE	Apolipoprotein E
MSCs	Mesenchymal Stem Cells
mTOR	Mammalian Target of Rapamycin
BDNF	Brain-Derived Neurotrophic Factor
GDNF	Glial Cell Line-Derived Neurotrophic Factor
iNOS	Inducible NOS
VEGF	Vascular Endothelial Growth Factor
